# Matrix Metalloproteinases in Neuropathic Pain and Migraine: Friends, Enemies, and Therapeutic Targets

**DOI:** 10.1155/2012/952906

**Published:** 2012-08-28

**Authors:** Shaheen E. Lakhan, Mihaela Avramut

**Affiliations:** ^1^Global Neuroscience Initiative Foundation, Los Angeles, CA 90210, USA; ^2^Verland Medical Communications, Pittsburg, PA 15201, USA

## Abstract

Matrix metalloproteinases (MMPs) constitute a family of zinc-dependent endopeptidases that mediate extracellular matrix turnover and associated processes, such as cell survival, growth, and differentiation. This paper discusses important functions of MMP in the normal and injured nervous system, focusing on the role played by these proteases in neurological pain syndromes, most prominently in neuropathic pain and migraine headaches. In the past decade, metalloproteinases emerged as key modulators of neuropathic pain, with MMP-9 acting as an initiator of the neuropathic cascade. Increased MMP activity was detected in migraine patients, independent of aura, in tight association with metabolic derangements. The therapeutic implications of MMP inhibition are considered in the context of neurogenic pain regulation.

## 1. Background

Matrix metalloproteinases (MMPs) are a family of over twenty zinc-dependent endopeptidases that play essential roles in a wide range of proteolytic processes. Their first-recognized and most prominent function is in cleaving components of the extracellular matrix (ECM). Additional substrates identified in recent years include other proteinases, chemotactic factors, growth factors, cell surface receptors, and cell adhesion molecules [1–3]. Acting upon all these substrates allows metalloproteinases to influence a multitude of processes, from cellular differentiation and migration to signaling, survival, and apoptosis (Table 1). Although most MMPs are secreted molecules, several transmembrane and glycosylphosphatidylinositol-anchored membrane proteins are also included in this family. According to their structure and substrate specificity, MMPs are categorized as collagenases (MMP-1, MMP-8, MMP-13, and MMP-18), gelatinases (MMP-2 and MMP-9), stromelysins (MMP-3, MMP-10, and -11), membrane-type MMPs (MMP-14 or MT1-MMP, MMP-15 or MT2-MMP, MMP-16 or MT3-MMP, MMP-17 or MT4-MMP, MMP-24 or MT5-MMP, and MMP-25 or MT6-MMP), and other MMPs [3].

In accordance with MMP involvement in tissue turnover, their activity undergoes tight regulation through multiple mechanisms, including transcription and translation of proprotease genes, and proform activation. Although most members of the protease family are not expressed constitutively, they are rapidly upregulated when cytokines, chemokines, growth factors, ECM components, and other transcriptional regulators act upon the cell [3, 4]. Most MMPs are translated as inactive precursors that subsequently require conversion to active enzymes under the influence of factors such as other MMPs, serine proteases, or free radicals. Termination of MMP activity relies on several mechanisms, including the action of four physiological tissue inhibitors of metalloproteinases (TIMPs) (TIMP-1, -2, -3, and -4).

The role of gelatinases in the nervous system has been the focus of numerous research studies, in part due to the availability of gelatinase zymography. While MMP-2 is constitutively expressed in many tissues, MMP-9 is highly inducible. After secretion, MMP-9 and MMP-2 are found in the ECM, cerebrospinal fluid, and serum. MMP-9 and MMP-2 are activated by other proteases. For instance, MMP-9 is activated by plasminogen/plasmin and MMP-3, while MMP-2 is activated by MMP-14 [5]. Using real-time polymerase chain reaction, Nuttall and collaborators determined that some subsets of leukocytes and central nervous system (CNS) cell types express transcripts for most MMP family members [6]. This finding suggests that all, or most, cell types have the ability to produce a spectrum of MMPs, but there are likely processes for selective expression in pathologic states.

MMPs are important for the structural development and maintenance of the nervous system. Studies employing knockout mice have shown that a deficiency in MMP-14 leads to smaller cranium, and an absence of MMP-19 results in abnormal cerebellar development. In the *Xenopus* eye, MMPs participate in the extension of optic nerve axons and in their guidance to the optic tectum [7–9]. In addition, studies conducted in normal rodents revealed that MMP-9 and MMP-24 are expressed in a similar pattern as TIMP-2 and -3 and appear to participate in vascularization, axonal growth, and neural plasticity [10–12]. Metalloproteinase activity appears to play a role in myelination, possibly by virtue of the need for ECM remodeling during oligodendrocyte processes extension. Both MMP-9 and MMP-12 were elevated in myelinating tracts from postnatal days 3 to 21, in parallel with developmental myelination [13]. Mice deficient in MMP-9 and MMP-12 exhibited impaired myelin formation. 

At present, evidence exists for a crucial physiological role of MMPs in the normal functioning of the adult CNS, especially in the regulation of synaptic plasticity, learning, and memory [2, 14]. These enzymes were shown to participate in hippocampal synaptogenesis and long-term potentiation (e.g., MMP-3 and MMP-9) [15, 16]. The proposed substrates that underlie MMP effects in neural development, maintenance, and function include regulation of ECM components, growth factors (such as insulin-like growth factor-1), proneurotrophins, receptors, and adhesion molecules [3].

When the nervous system is injured, MMP transcription and synthesis increase in several cell types to promote local repair, remyelination, regeneration, and even angiogenesis [17–22]. The subsequent sections of this paper focus on the important roles played by several MMPs in the genesis of pain sensation associated with nervous system pathological states, with emphasis on neuropathic pain and migraine.

## 2. MMP Modulators of Neuroimmune ****Interactions and Nociception 

Numerous studies revealed intriguing roles for MMPs in the pathogenesis of nervous system disorders, from trauma to stroke, Alzheimer's disease, multiple sclerosis, and amyotrophic lateral sclerosis [23–27]. In these circumstances, several MMPs may become simultaneously elevated, in accordance with symptoms and neuropathological features. These observations led to the hypothesis that ordinarily beneficial metalloproteinases can become detrimental, possibly as a consequence of aberrant proteolysis when enzymatic expression becomes concurrent, extensive, and exaggerated, instead of limited to the injury site [13] or as a result of an alteration in MMP/TIMP balance. Given the roles MMPs play in neuroinflammation (by cleaving extracellular matrix proteins, cytokines, and chemokines), this abnormal expression pattern leads to phenomena such as opening of the blood-brain barrier (BBB) [5, 28–30], with ensuing edema and vascular leakage; invasion of neural tissue by blood-derived immune cells [31]; shedding of cytokines and cytokine receptors [32]; direct cellular damage in diseases of the peripheral and central nervous systems. It is now accepted that MMPs can directly or indirectly kill neurons by multiple mechanisms, such as interfering with adherence and tumor necrosis factor (TNF) receptor apoptosis cascade, or producing neurotoxic chemokine species [33, 34].

One of the most exciting developments in the field of MMP research is a new appreciation of the role played by these enzymes in nociception and hyperalgesia. Although the evidence for a direct influence of MMPs on peripheral nociceptors remains scarce, MMP may prove important in the generation of pain induced by inflammation and nerve lesion via their complex relationship with cytokines, chemokines, growth factors, and adhesion molecules to which nociceptors are responsive. Interleukin-1*β* (IL-1*β*), TNF, and nerve growth factor, for example, elicit action potentials by amplifying sodium and calcium currents at the nociceptor peripheral terminal [35]. After neural damage, these same inflammatory mediators are released by peripheral immune cells and microglia in the spinal cord and contribute to neuropathic pain by activating nociceptive neurons. 

Recent studies demonstrated that MT5-MMP (MMP-24), expressed by peptidergic nociceptors in dorsal root ganglia (DRG), modulates cell-cell interactions between nociceptive neurites and mast cells and represents an essential mediator of peripheral thermal nociception and inflammatory hyperalgesia [36]. Mutant mice deficient in this MMP had increased sensitivity to noxious thermal stimuli under basal conditions. Consistently, mutant peptidergic sensory neurons hyperinnervated the skin, and this phenotype correlated with changes in the regulated cleavage of the cell-cell adhesion molecule N-cadherin. In contrast to basal nociception, MMP-24(−/−) mice did not develop thermal hyperalgesia during inflammation, which could reflect alterations in N-cadherin-mediated cell-cell interactions between mast cells and sensory fibers. These studies provided evidence that absence of an MMP family member leads to altered neuroimmune interactions and nociception.

Christianson and colleagues [37] recently reported that spinal MMP-3 is also implicated in the coordination of spinal nociceptive processing and inflammatory hyperalgesia via a spinal TNF-dependent mechanism. The time-dependent upregulation of MMP-9 and MMP-2 after peripheral nerve injury has been associated with both detrimental and beneficial effects on recovery [2, 38]. Produced by both neurons and glia, MMP-2 and MMP-9 mediate pain hypersensitivity by initiating IL-1*β* cleavage and microglial and astrocytic activation [35, 39]. Matrix metalloproteinase 9 has recently emerged as an essential component of the Schwann cell signaling network during sciatic nerve regeneration [40]. The MMP-9/TIMP-1 axis plays an important role in guiding the myelinating Schwann cells differentiation and the molecular assembly of myelin domains during nerve repair. The reported MMP-dependent regulation of sodium channels may provide a basis for therapeutic intervention in sensorimotor pathologies and pain.

### 2.1. Neuropathic Pain

Neuropathic pain (NP), one of the most difficult clinical pain syndromes to treat, represents an unpleasant somatosensory experience that results from injuries to the peripheral nervous system (PNS) or the CNS (e.g., spinal cord or thalamus). These injuries have various causes, such as accidental trauma, major surgeries (e.g., amputation), stroke, diabetes, viral infection (e.g., HIV), and chemotherapy (e.g., paclitaxel and vincristine). The syndrome manifests with both spontaneous and evoked pain. Excessive pain in response to noxious stimuli (hyperalgesia) and pain elicited by normally innocuous stimuli (allodynia) represent distinct symptoms. Initial investigations revealed peripheral and central neural mechanisms underlying this syndrome. Peripheral sensitization occurs when primary afferent nociceptive neurons exhibit increased responsiveness to external mechanical or thermal stimuli at the original site of injury/inflammation mediated by proinflammatory cytokines and other molecules [41, 42]. Central sensitization involved increased excitability of dorsal horn neurons resulting in increased synaptic strength and enlargement of their receptive fields beyond the original site of injury/inflammation. This process leads to persistent and extended pain [43]. In addition, recent studies revealed intriguing nonneuronal substrates for NP generation. Microglial and astrocytic activation, as well as glial production of proinflammatory cytokines under the control of mitogen-activated protein kinases (MAPKs) such as extracellular signal-regulated kinase (ERK), constitute examples of mechanisms currently explored in the quest for therapeutic targets [44]. Proteases, among them MMPs, have emerged as important modulators associated with non- neuronal pain pathways. Thus far, the main MMPs that have been implicated in the generation and maintenance of NP are MMP-2 and MMP-9, but other enzymes (e.g., MMP-3, MMP-24) may be involved. The temporal and cellular profiles of MMP expression in the injured spinal cord have been studied and reviewed extensively [45]. While MMP-2 is constitutively expressed in the normal brain and spinal cord, MMP-9 is upregulated following injury.

Analyses of biopsy tissue underscore the importance of MMP in the pathogenesis of neuropathy. These endopeptidases are involved in tissue destruction and infiltration by immune cells in multiple sclerosis [46, 47] and Guillain-Barré syndrome (GBS). In GBS, an acute inflammatory demyelinating neuropathy is characterized by humoral and cellular immune dysfunctions, the immune reaction associated with increased proinflammatory cytokines (e.g., TNF-*α*), decreased anti-inflammatory cytokines (e.g., TGF-*β*1), and increased MMP-9 [48]. Such abnormalities favor a central pathogenetic mechanism: the adhesion to and transmigration across endothelium of immune cells. 

Elevated MMP-2 and MMP-9 immunoreactivity was found in nerve tissue in chronic inflammatory demyelinating polyneuropathy (CIDP) and nonsystemic vasculitic neuropathy (NSVN), compared to noninflammatory neuropathies (NINs) [49]. The similar increase of MMP-2 and MMP-9 in both demyelinating (CIDP) and nondemyelinating (NSVN) neuropathies raises doubts regarding a potential primary role of MMPs in demyelination. T cells constituted the predominant source of MMP-2 and MMP-9 in CIDP and NSVN. Stromal cells of the perineurium and epineurium were an additional source of MMP-2 in NSVN, but not in CIDP. Expression of MMP-3 and MMP-7 was not detectable in CIDP or NSVN. The levels of MMP-2 and MMP-9 did not correlate with clinical disease activity or electrophysiologic measurements.

Gurer and collaborators [50] studied nerve biopsy sections from patients with NSVN and systemic vasculitic neuropathy (SVN) and compared them with controls belonging to subjects with noninflammatory neuropathy. Expression of MMP-9, but not MMP-2, was increased in perivascular inflammatory infiltrate in nerve tissues of vasculitic neuropathy patients. This MMP-9 expression correlated positively with immunostaining of CD8+ T cells. Patients with NSVN exhibited enhanced MMP-9 immunoreactivity compared to SVN. These results suggest a pathogenic role for MMP-9 secreted from CD8+ cells in vasculitic neuropathy.

In systemic lupus erythematosus (SLE), a peripheral neuropathy is often seen. Its precise pathogenetic mechanisms remain unclear and possibly involve ischemic nerve damage due to vasculopathy and vasculitis of the nutritional vessels. Abnormal MMP levels have been reported in the serum of SLE patients [51]. When the expression of MMP-1, -2, -3, -9, -10, and -13 and their tissue inhibitors (TIMP-1 and -2) was analyzed in sural nerves from SLE patients in comparison to normal controls [52], all MMPs could be detected within blood vessel walls from SLE nerves. In controls, MMP-3 and MMP-9 were not detected. Small and large nutritional vessels in the epineurium were immunoreactive for MMPs and TIMPs. Mononuclear cells, which expressed MMP-1, -3, -10, -13, and TIMP-1, were also observed in most of the SLE nerves, mostly around epineurial blood vessels, but only occasionally in controls. These results led to the conclusion that MMP expression in mononuclear cells may be related to leukocyte trafficking through the vessel walls. The upregulation of MMP-3 and MMP-9 in particular, within the vessel walls, could underlie the vascular damage seen in SLE and the resulting chronic combined axonal and demyelinating type of neuropathy frequently found in these patients.

Other immunohistochemical investigations [53] performed on nerve samples of inflammatory and noninflammatory polyneuropathic subjects revealed perineurium and endothelium MMP-2 positivity in all tissue sections, with a specific upregulation of stromal MMP-2 in chronic inflammatory demyelinating polyneuropathy (CIDP), and even higher levels in vasculitic neuropathies. Cells positive for MMP-9 were detected in vessel walls, infiltrates, epineurium, and endoneurium of vasculitic neuropathies. In CIDP, MMP-9-positive cells were prominent in vessel walls. Double staining indicated that the infiltrating cells were T cells and macrophages. Taken together, these findings point to an important role for MMP-9 in inflammatory peripheral neuropathy, most likely in connection with inflammatory cell invasion.

**Peripheral Nerve Injury-Induced Pain**

**Figure 1:**
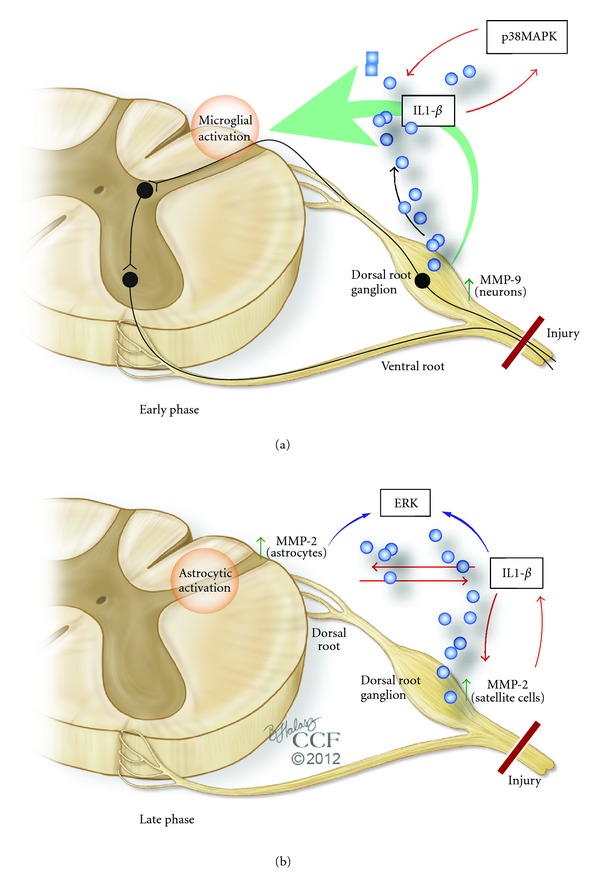
Changes in gelatinases (MMP-9 and MMP-2) and critical substrates during distinct phases of neuropathic pain induced by spinal nerve injury. In the first several days after spinal nerve injury in animal models (*early phase*), MMP-9 becomes upregulated in the dorsal root ganglion (DRG) neurons, where it is needed for IL-1*β* cleavage and activation. From the DRG neuronal soma, the gelatinase is transported to the dorsal horn central terminals, to activate microglia (via activation of a feedback loop between a mitogen-activated protein kinase, p38 MAPK, and IL-1*β*). In contrast, MMP-2 is induced and persists in DRG satellite cells and spinal cord astrocytes in the late phase of neuropathic pain generation (from a week to months after injury). This MMP is important in activating IL-1*β*, extracellular-regulated kinases (ERK), and astrocytes. A positive feedback also exists from IL-1*β* to both ERK and MMP-2 (which increases MMP-2 expression). Abundant evidence indicates that activation of microglia and astrocytes in the dorsal horn represents an essential amplification mechanism leading to neuropathic pain in the setting of spinal cord or nerve injury.

In animal models, at least two phases of NP can be distinguished: an early phase, in the first days (when the pain develops), and a late phase, in the subsequent weeks and months (see Figure 1). Several cellular and molecular mechanisms have been shown to contribute to pain generation in each of these phases. It is important to recognize, however, that current animal models, predominantly symptomatic, may not be relevant to the timing of the clinical neuropathic pain syndrome, which persists after the healing of the lesion and can occur late after injury.

Myelin protects A-*β* afferents from ectopic hyperexcitability and nociception triggered by innocuous mechanical stimuli. After mechanical damage to the axon, Schwann cells release MMP-9, initiating macrophage infiltration and degradation of myelin basic protein [38, 54]. Exposure of the bare axon leads to increased sodium channel expression and ectopic hyperexcitability of afferents [55]. As a result, action potentials outlast the stimulus, contributing to central sensitization. Proinflammatory cytokines such as interleukin (IL)-1*β*, released after nerve injury, play essential roles in NP sensitization [39, 56]. Signaling pathways that involve growth factors (e.g., Neuregulin-1), MMP (e.g., MMP-9), and several chemokines enable direct communication between injured primary afferents and microglia [57]. While rapid microglial activation plays an important role in the induction and early-phase development of NP, delayed and persistent astrocyte activation helps to maintain and develop late-phase NP [44]. Astrocyte activation (as evidenced by upregulation of the astrocyte markers glial fibrillary acidic protein, GFAP, and S-100) remains at the peak level even in the late phase. The phosphorylated ERK (pERK) undergoes dynamic changes in different spinal cell types at different times after nerve injury. Inhibition of this pathway in the spinal cord during either the early or the late phase effectively diminishes NP. 

Rodent models of NP with spinal nerve ligation at the L5 level constitute a valuable experimental paradigm for the study of NP pathogenesis. After lesioning, a rapid (less than one day) but transient (more than three days) upregulation of MMP-9 in the DRG was noted [36]. Administration of MMP-9 via intrathecal injection produced mechanical allodynia. Conversely, intrathecal administration of MMP-9 inhibitors (such as TIMP-1 or synthetic compounds) reduced NP in the early phase, without systemic effects. It was hypothesized that the mechanism of MMP-9-induced neuropathic pain involves IL-1*β*, as MMP-9 knockout mice lose the marked IL-1*β* activation (cleavage) in the DRG in the early-phase (day one) postnerve injury. The injury-induced spontaneous discharge in sensory neurons might release MMP-9 and pro-IL-1*β* into the ECM, where MMP-9 cleaves pro-IL-1*β* to produce active IL-1*β*. The active IL-1*β*, in turn, generates hyperexcitability by acting on adjacent nociceptive neurons. Behavioral studies conducted by Zhang and collaborators [45] indicated that blocking IL-1*β* with a neutralizing antibody suppresses neuropathic pain symptoms induced by MMP-9 or by nerve injury. In this early phase, MMP-9 from DRG rapidly reaches the central terminals in the dorsal horn and activates microglia [44]. Here, the resulting low concentrations of metalloproteinase are sufficient for microglial activation but do not produce demyelination and apoptosis. Taken together, these data support an important role of MMP-9 and IL-1*β* in neuropathic pain development via IL-1*β* production and microglial activation.

In similar models of spinal nerve lesions, an upregulation of MMP-2 is observed. This increase exhibits a distinct pattern. Unlike the rapid and transient MMP-9 surge, MMP-2 upregulation is delayed (over a week) and persistent (more than three weeks) and occurs predominantly in small satellite cells surrounding DRG neurons. Instead of a low level in the spinal cord terminals, MMP-2 exhibits a persistent induction in astrocytes at this level [41]. Interestingly, these astrocytes, and the satellite DRG cells, also undergo persistent increases in GFAP and pERK after nerve injury that contributes to NP establishment. 

While injecting exogenous MMP-2 induces pain, studies employing small synthetic inhibitors, an endogenous peptide inhibitor (TIMP-2), and small interfering RNA have shown that MMP-2 inhibition effectively reduces late-phase NP. In addition, MMP-2 inhibition suppresses pERK induction in spinal cord astrocytes. These and other studies suggest that, at this stage, MMP-2 plays important roles in IL-1*β* cleavage and activation and spinal astrocytes activation [5]. 

The emerging scenario, therefore, features MMP-9 central in promoting NP generation through IL-1*β* cleavage and microglial activation in the early stage, while MMP-2 maintains pain through IL-1*β* cleavage and astrocyte activation in later stages. Interestingly, studies conducted in a rodent model of sciatic nerve injury [58] showed that MMPs were beneficial in the early response to injury, by promoting regeneration of peripheral nerve. MMP-2 was transported to the axonal growth cone, further suggestive of a beneficial role in peripheral nerve repair [59]. An increase in this metalloproteinase accompanied the analgesic effects of a neuronal nitric oxide synthase inhibitor [60]. Overall, the evidence points to dual roles for gelatinases after peripheral nerve injury: (1) mediators of neuroinflammation and pain and (2) promoters of analgesia and repair [40, 61].

Other MMPs are implicated in nociceptive processing after nerve injury. An upregulation of MMP-3 in the DRG participates in triggering paclitaxel-induced peripheral neuropathic pain [62]. Mice lacking MT5-MMP (MMP-24) did not develop NP with mechanical allodynia after sciatic nerve injury, despite normal responses to noxious stimuli [63], indicating that this MMP is also a key player in NP pathogenesis.

Microarray studies underscore the importance of MMPs in NP (as opposed to exclusively inflammatory pain). Tissue inhibitor of metalloproteinase 1 (TIMP-1) is one of four genes relatively increased in the spinal cord of animals with neuropathic, but not inflammatory, pain [64].

**Spinal Cord Injury-Induced Pain**

Spinal cord injuries leave as many as 85% of patients with chronic neuropathic pain in dermatomes at, below, or above the injury level [65]. The essential mediators of neuroinflammation are similar in peripheral nerve injury and spinal cord injury-induced pain [66]. However, while MMPs play essential roles in the development of NP in peripheral injury, their contribution to spinal cord injury induced pain remains to be elucidated. Cannabinoid receptors (CBRs) and transient receptor potential vanilloid 1 (TRPV1, a calcium-permeable nonselective cation channel) may provide a link between the MMP network and nociceptive mechanisms. Cannabinoid agonists diminish thermal hyperalgesia after injury, while CBR stimulation induces TIMPs [67, 68], thereby inhibiting MMPs. Activation of TRPV1R plays a role in heat shock-induced MMP-1 expression in human epidermal keratinocytes [69], and the same channels are upregulated in animals demonstrating NP following spinal cord injury [70].

### 2.2. Migraine

A common neurological condition, migraine manifests with recurrent attacks of severe, throbbing head pain sometimes preceded by visual, somatosensory, or motor neurological symptoms (migraine with aura) or not (migraine without aura), or exhibiting atypical presentations (migraine “variants”). The pathophysiology of this debilitating headache disorder remains incompletely understood. After alternating between purely neuronal and predominantly vascular explanations for this puzzling disorder, researchers are now recognizing the complexity of its likely neurovascular pathogenetic mechanisms including cortical neuronal hyperexcitability, hypoperfusion, transmembrane ionic dysfunction, vasoactive substances and neurotransmitter abnormalities, neurogenic inflammation, and cortical spreading depression (CSD) [71, 72].

CSD is a slowly propagating wave of depolarization that presumably underlies aura symptoms and is perhaps clinically silent in migraine without aura. Through various mechanisms, CSD increases the expression of many genes, most prominently MMPs, cyclo-oxygenase 2 (COX-2), TNF-*α*, and IL-1*β*. Upregulated metalloproteinases (e.g., MMP-9) alter BBB permeability, thereby allowing potassium, nitric oxide, adenosine, and other factors to reach and sensitize perivascular trigeminal afferent endings in the dura mater [73–75].

Interestingly, clinical studies demonstrated higher circulating MMP-2 and MMP-9 activity in migraineurs, with or without aura, compared with unaffected controls [76, 77]. In some studies, migrainous patients showed higher MMP-9 plasma levels during headache attacks than in asymptomatic periods, regardless of aural status [78], but other reports contradict this finding and suggest MMP-3 is decreased at the ictal stage [79]. When measured in isolation, MMP-9 levels appears to be the same for migraine patients with and without aura [76]. In patients suffering from migraine without aura, however, the ratio of MMP-9 to TIMP-1 was increased compared to patients with aura [77], reflecting a potential distinct pathophysiological mechanism. Highly significant increased MMP activity in migraine patients, independent of aura symptoms, was tightly associated with migraine-related hyperinsulinemia and atherogenic lipid alterations [80].

## 3. Therapeutic Significance 

The tetracycline drug minocycline, proposed for the treatment of various types of neurological diseases, happens to be an inhibitor of MMP-9 [81]. Various pharmacological metalloproteinase inhibitors (MPIs) have been developed that target inflammation-associated disorders such as arthritis, atherosclerosis, multiple sclerosis, and cancer [82]. Whether they will become part of the therapeutic armamentarium in these disorders remains to be established, as many phases I, II, and III clinical trials were disappointing, especially for oncological applications. Meanwhile, the complexity of NP pathogenesis renders therapeutic intervention difficult. As many studies suggested, inhibition of MMP-9 or MMP-2 may constitute a useful strategy for the prevention and treatment of NP. Theessential role of MT5-MMP in the development of dermal neuroimmune synapses suggests that this metalloproteinase can also become a target for pain control.

In animal studies, intrathecal or intraperitoneal delivery of MPI shows significant promise in attenuating allodynia and interfering with neuropathogenetic cascades. Acute and long-term therapy with GM6001 (a broad-spectrum MPI), for example, protected from injury-induced MBP degradation, caspase-mediated apoptosis, and macrophage infiltration in the spinal nerve as well as inhibited astrocyte activation in the spinal cord [38]. The effect of GM6001 therapy on attenuation of mechanical allodynia was robust, immediate, and sustained through the course of the spinal nerve lesion. Moreover, the estimated temporal profile of MMP expression raises the possibility of tailoring NP treatment per stage. The endogenous inhibitor TIMP-1, for example, is 1,000 times more potent than morphine in alleviating pain (and exhibits a longer duration of pain suppression) but only works in the early phase (e.g., first day) [5]. Given their high potency in suppressing neuropathic pain at different phases, TIMPs hold promise for treating neuropathic pain. Unfortunately, selecting the specific type of inhibitor, and the adequate time for its administration, can become difficult task in clinical settings.

Synthesizing selective gelatinase inhibitors represents a challenge, due to the structural similarities between MMP family members [45, 83]. First-generation synthetic competitive MPIs (e.g., broad-spectrum peptidomimetics batimastat and marimastat) lacked selectivity and induced musculoskeletal injury [84]. More selective inhibitors have been developed, such as prinomastat [85], that nevertheless can target other, nongelatinase MMPs (reviewed in [45]). The ability of tetracyclines to inhibit MMPs constitutes the basis for their use in treating periodontitis (doxycycline) and in animal models of neurological diseases (minocycline, a neuroprotective MMP-9 inhibitor). Mechanism-based inhibition, during which the inhibitor-enzyme complex undergoes a nonreadily reversible conformational change, was first achieved with SB-3CT, a selective inhibitor of MMP-2 and MMP-9, and reversible MMP-14 inhibitor. The compound has demonstrated efficacy in spinal cord injury, stroke, and cancer metastasis [86, 87]. Numerous derivatives of SB-3CT have been designed to circumvent its rapid and extensive metabolism; these compounds display selective, slow-binding MMP-2 or MMP-9 selectivity [88]. 

The advent of all these agents opens new perspectives for treating pain. The study of MMP-inhibiting compounds remains a prolific field in academic and industrial settings, with phase I trials exploring the efficacy of novel inhibitors in pain syndromes (e.g., orofacial pain) currently underway. One possible phenomenon underlying the conflicting results of clinical trials is that MMP inhibitors may also influence other endopeptidases or MMP-mediated processes (e.g., TNF release and shedding of the IL-6 receptor), leading not only to therapeutic effects [89], but also to negative outcomes. It was suggested, for example, that broad-spectrum MMP inhibitors affect TNF release and can exacerbate liver damage [90]. It also became evident that administration of MMP inhibitors may induce joint pain in some patients, further underscoring the complexity of MMP regulation [89]. To ensure therapeutic efficacy, selective compounds should be employed, such as MMP-9 or MMP-2 small molecule inhibitors, peptide inhibitors, monoclonal antibodies, and small interfering RNA (siRNA), with careful consideration of temporal and spatial patterns of MMP expression after injury. Dual inhibition of MMP-9 and MMP-2 may be used to treat neuropathic pain at distinct phases. Caution should be exerted, however, when long-term treatment is required: minocycline, for example, worsened the condition of some ALS patients when given at late stages [91], despite showing beneficial effects in newly diagnosed patients [92].

Nonpharmacological therapies may provide additional ways of influencing MMP activity. Studies investigating the analgesic effect of electroacupuncture in a rat model of NP showed a reduction in allodynia, GFAP, MMP-9/MMP-2, and proinflammatory cytokines in treated animals [93], suggesting that the analgesic effect may be partly mediated via inhibition of glial activation and MMPs.

## 4. Conclusion and Future Directions 

In breaking the “shell” of the ECM and BBB and contributing to neuroinflammation and pain generation, metalloproteinases reveal themselves as valuable targets for pain relief. Continuous research efforts aim to reveal their precise roles in neuropathic and migraine cascades. Notoriously difficult to manage, patients with NP often derive little comfort from current, neurotransmitter-centered therapies that elude the pathogenetic mechanisms of the disorder and do not differentiate between its phases. Studies indicate that MMP-9 inhibition can be employed to treat established NP and even to prevent postsurgical or posttraumatic NP. Moreover, elucidating the MMP- and TIMP-dependent pathways in migraine, and clarifying the effects of MMP inhibitory drugs, will prove beneficial in migraine therapy. More attention must be devoted to exploring MMP involvement in pain syndromes caused by spinal cord injury and neuromuscular diseases. Achieving a thorough knowledge of MMP substrates in the nervous system and targeting specific enzymes with monoclonal antibodies or siRNA will help reduce the side effects of therapeutic inhibition.

MMPs contribute to numerous mechanisms of secondary pathogenesis following nervous system injury, chiefly through their involvement in neuroinflammation, but also—often paradoxically—by supporting regeneration and vascular remodeling processes. Any therapeutic attempt to manipulate MMP activity has to take into consideration this duality of function, in addition to the complex metalloproteinase network interrelationships.

## Figures and Tables

**Table 1:** Matrix metalloproteinases modulate tissue structure and cellular activities by participating in complex biological processes.

Developing tissue (normal and injured)	Adult tissue (normal and injured)
(i) Embryogenesis and morphogenesis (ii) Cell migration, differentiation, and death (including neuronal migration, dendritogenesis, synaptogenesis, and myelination)	(i) Normal tissue maintenance and remodeling (ii) Wound healing (iii) Angiogenesis (iv) Neuroplasticity and neurorepair (v) Leukocyte adherence and transmigration (vi) Release of biologically active molecules (e.g., growth factors, inflammatory modulators) (vii) Vasogenic edema (viii) Tumor cell survival and metastasis (ix) Cell growth inhibition

## Abbreviations

BBB: Blood-brain barrier
CBR: Cannabinoid receptors
CNS: Central nervous system
CSD: Cortical spreading depression
DMD: Duchenne muscular dystrophy
DRG: Dorsal root ganglion
ECM: Extracellular matrix
ERK: Extracellular signal-regulated kinase
GFAP: Glial fibrillary acidic protein
IL: Interleukin
MAPK: Mitogen-activated protein kinase
MMP: Matrix metalloproteinases
MPI: Metalloproteinase inhibitor
NP: Neuropathic pain
PNS: Peripheral nervous system
siRNA: Small interfering RNA
TIMP: Tissue inhibitor of metalloproteinases
TNF: Tumor necrosis factor
TRPV1: Transient receptor potential vanilloid 1.
